# Dendrite Pattern Formation of Sodium Chloride Crystal

**DOI:** 10.3390/ma14164434

**Published:** 2021-08-07

**Authors:** Nobuhiko J. Suematsu, Junpei Iwamoto, Yuya Ishii, Akira Yamamoto

**Affiliations:** 1 Graduate School of Advanced Mathematical Sciences, Meiji University, 4-21-1 Nakano, Tokyo 164-8525, Japan; 2 Meiji Institute of Advanced Study of Mathematical Sciences (MIMS), Meiji University, 4-21-1 Nakano, Tokyo 164-8525, Japan; 3 School of Interdisciplinary Mathematical Sciences, Meiji University, 4-21-1 Nakano, Tokyo 164-8525, Japan; ev30075@meiji.ac.jp (J.I.); ev40034@meiji.ac.jp (Y.I.); ev60040@meiji.ac.jp (A.Y.)

**Keywords:** pattern formation, dendrite, sodium chloride, crystallization

## Abstract

A variety of crystal structures is found in nature, not only equilibrium structures reflecting molecular structures, but also non-equilibrium structures which depend on the physicochemical conditions occurring during the crystal growth. In this paper, we focus on the dendrite structure of sodium chloride (NaCl) formed by the simple evaporation of an aqueous NaCl solution. The characteristics of the growth structures were measured as a function of the initial concentration of NaCl. In addition, the crystal growth process was measured using optical microscopy. As a result, the growth rate was not constant but was found to oscillate over time and synchronize with the wetting of the crystal. Our observations indicate that dendrite structures are more easily generated at higher initial concentrations. The detailed mechanism for dendrite pattern formation is still under investigation.

## 1. Introduction

The variety of crystalline structures reflects the molecular structures and types of chemical bonds. Equilibrium crystal structures are obtained by minimizing energy. On the other hand, when crystals form under a far-from-equilibrium condition, their structure depends not only on the energy loss, but also on the dynamics of crystal growth. In the latter case, the shape of crystals can be dramatically different from those obtained at equilibrium. The most famous example is the snow flake which is a dendritic shape of ice. The growth structure depends on the environmental physicochemical conditions [1]. The structure of a snow flake can be explained by invoking the Mullins–Sekerka instability, that is, the grow speed depending on the curvature of the surface [2,3]. Other examples are dendrites such as copper dendrites [4] and the electrochemical deposition of zinc [5,6,7]. The formation of these dendrite patterns is understood in terms of diffusion-limited aggregation [8] and recently several works have succeeded in reproducing such pattern formations numerically [9,10].

In this paper, we focus on the dendrite structure of a sodium chloride (NaCl) crystal. The dendrite growth of a salt crystal surface [11,12,13,14], lifting up of the crystal with the evaporation of a NaCl aqueous solution [15], and an upward crystal growth on walls [16] have been reported. These examples are a simple experimental setup that is just dry NaCl aqueous solution. Nevertheless, varieties of the complex structures are generated. It is due to the structure formation occurring under far from equilibrium and, thus, the structure is not just a lowest energy structure, but is determined through the growth speed. In order to uncover the mystery behind such a complex structure formation, we systematically changed the physicochemical conditions such as the evaporation rate of the aqueous solvent, the initial concentration of NaCl, and the surface condition of the solid glass, and observed the NaCl crystal structures. To estimate the characteristics of the structure, we measured the fractal dimension [4]. Based on our observations, we suggest a preliminary explanation for the growth process of NaCl dendrites.

## 2. Materials and Methods

Sodium chloride (NaCl) was purchased from Wako Pure Chemical Corporation (Kyoto, Japan). Water was purified with Milli-Q filtering system. A large-sized slide glass (S9224; 76 mm in height, 52 mm in width, and 1.2 mm in thickness) and a slide glass with cover glass (MUR-500; 76 mm in height, 26 mm in width, and 1.2 mm in thickness) were bought from Matsunami Glass Ind., Ltd. (Osaka, Japan).

**Figure 1:**
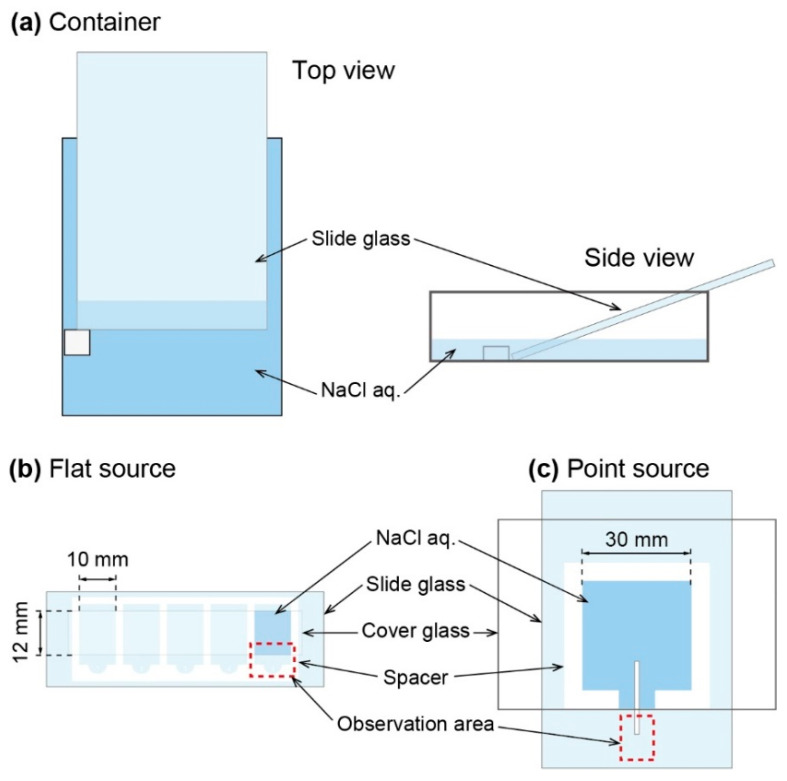
Illustration of experimental setup. (**a**) Edge of a slide glass was soaked into NaCl aqueous solution (5.4 M). (**b**) An aqueous solution of NaCl (2.0–5.4 M) was added in thin space between a slide glass and a cover glass, whose gap was 0.07 mm. (**c**) An aqueous solution of NaCl (2.0–5.0 M) was put on a slide glass with silicon sheet (thickness: 0.5 mm). Elongated filter paper (width: 2 mm) was put on the glass. Aqueous solution was covered with another slide glass.

Three types of observation systems were constructed, i.e., (i) open container, (ii) flat source, and (iii) point source. The open container system composed of a plastic container (110 mm in length, 80 mm in width, and 33 mm in height), a piece of silicon as a stopper, and a large-sized slide glass. The slide glass was put on a container with 20 degrees as the angle against the bottom of container. To keep the glass, a piece of silicon was pasted to the bottom of the container (Figure 1a). Then, aqueous solution of NaCl (5.4 M) was added into the container. The volume of the NaCl aq. was 35 mL. The growth of NaCl crystal on the slide glass was monitored using digital camera (EOS Kiss, Canon, Tokyo, Japan). Photograph was taken every 30 min. Flat source system was realized with a slide glass with cover glass, whose space was 10 mm in width, 12 mm in height, and 0.07 mm in thickness. An aqueous solution of NaCl (2.0–5.4 M) was added into the space between glasses (Figure 1b). The growth process was observed by optical microscope (Eclipse TS100, Nikon, Tokyo, Japan). Point source system was constructed by two large-sized slide glasses, a silicon sheet as a spacer (0.5 mm in thickness), and an elongated filter paper (2 mm in width). An aqueous solution of NaCl (2.0–5.0 M) was sandwiched by two large-sized glasses with the spacer (Figure 1c). Crystal growth of NaCl was monitored with digital microscope (VHX-5000, Keyence, Osaka, Japan).

Image data were analyzed on PC using image analysis software, “ImageJ”. We measured several parameters such as brightness, growth rate, and fractal dimension of the crystal. Local brightness of the crystal was measured by averaging the pixels value. The growth rate was estimated by the edge position of the crystal over time, which was determined by the boundary of bright and dark regions. At first, we set an analysis line as was orthogonal to the interface of the crystal and obtained the pixels value on the line. The pixels value on the crystal was high and on the background was low, because we used dark background. Therefore, the pixels value drastically changed at the edge of crystal. Therefore, we could obtain the position of the crystal edge by analyzing on the line and calculating the growth rate of the crystal on the analysis line. In order to estimate fractal dimension of the crystal shape, we measured pixel number on the perimeter of crystal with change in the resolution of the images. We used the power index as fractal dimension. The power index was obtained by logarithmic plot of the perimeter against the resolution.

## 3. Results

**Figure 2:**
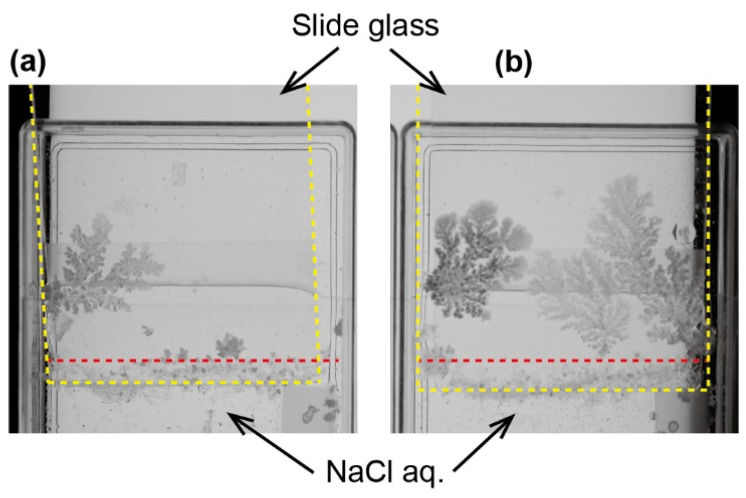
Dendritic crystal growth of NaCl on a large-sized slide glass. Contact angle of water droplet on the glass plate was (**a**) 65° and (**b**) 50°. Red and yellow broken lines indicate three-phase contact line and contour of slide glass, respectively.

The dendritic crystal structure of NaCl was generated on a glass surface in the open container system (Figure 2). Upon evaporating the solvent, small crystals of NaCl were induced on the surface of the NaCl aqueous solution. The small crystal often reached and was pinned at the three-phase line by chance. Then, the crystal grew toward the opposite direction of the NaCl solution and its shape became dendrite. The possibility of the pinning of the small crystal and the growth rate of dendrite was higher and faster on the hydrophilic surface rather than the hydrophobic one (Figure 2).

**Figure 3:**
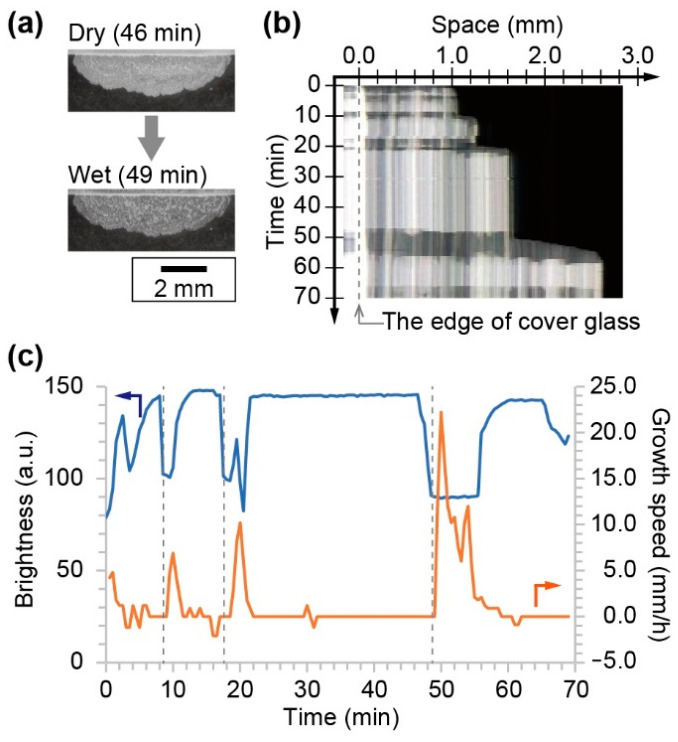
Observation results of flat source system. (**a**) Snapshots of dry and wet crystals. (**b**) Space–time diagram of crystal growth. (**c**) Time series of local brightness and growth speed of NaCl crystal. Blue and orange solid lines indicate brightness and growth speed, respectively. Broken lines indicate the timing of wetting the crystal.

The crystal growth toward the opposite direction of the source of the NaCl solution was also observed in the flat source system, where the NaCl solution was almost covered with glass. In this system, seed crystals were produced at the edge of the cover glass and in contact to the surface of the slide glass. From the seed crystal, a solid crystal of NaCl was produced on the slide glass. During the growth process, an alternation between a dry and a wet state was observed (Figure 3a). The dry crystal looked bright, while the wet one was dark. The space–time diagram indicated that the wetting and drying was repeated during the crystal growth (Figure 3b). The growth speed strongly depended on the local brightness at the arbitral position near the edge of the cover glass (Figure 3c). The time series of the brightness and growth speed indicated that the crystal growth occurred only at the wetting period. The interval of the wetting process became long concurrent with the increase in the size of the crystal.

**Figure 4:**
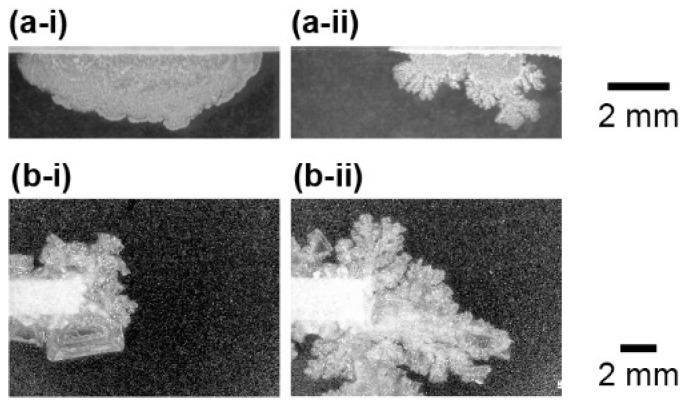
Shape of NaCl crystals formed on a glass surface. (**a**) Flat source system and (**b**) point source system. The concentrations of NaCl in the source solution were (**a-i**) 2.5 M, (**a-ii**) 5.0 M, (**b-i**) 3.0 M, and (**b-ii**) 5.0 M.

The shape of the NaCl crystal and growth speed strongly depended on the concentration of the source solution of NaCl. With a low concentration source, the growth speed of the crystal was slow and the shape was of round a form (Figure 4a-i,b-i). On the other hand, a high concentration solution produced dendrite structures (Figure 4a-ii,b-ii, and see also Video S1 in the Supplementary Materials) and its growth speed was faster than that in a low concentration source. This relationship was observed in both the flat and point source systems. However, the round shape was hard to observe in the point source system. It indicated that the dendrite pattern tends to be generated in the point source system.

**Figure 5:**
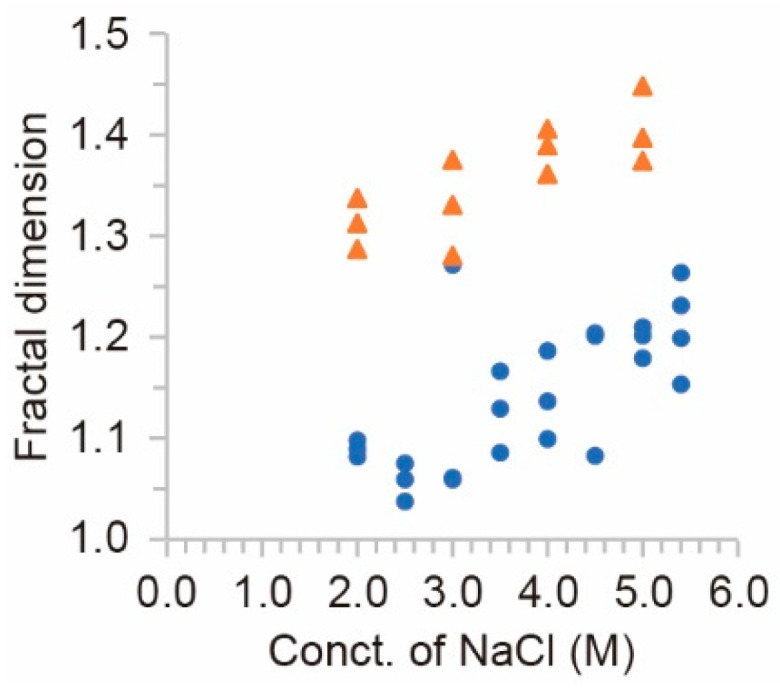
Fractal dimension of NaCl crystal. Blue circle are the results from flat source and orange triangles from point source.

In order to estimate the characteristics of the shape of crystals, the fractal dimension was measured for all the experimental observations. Here, the perimeter length of the NaCl crystals was measured with change in the resolution of the image. Then, the fractal dimension was measured by a logarithmic graph of perimeter and resolution, whose slope corresponds to the fractal dimension. As is shown in Figure 4, the shape of crystals became more complex through the higher concentration of source rather than the lower one. This relationship was quantitively supported by the fractal dimension depending on the concentration of the source aqueous solution. Namely, the fractal dimension tended to increase with the concentration of NaCl (Figure 5). In addition, for all concentration sources, the point system generated higher fractal dimension structures than the flat system.

## 4. Discussion

A crystal usually grows toward the source, namely, toward the solution phase. On the other hand, the NaCl crystal grew upward which was the opposite direction of the NaCl solution. The mechanism for this interesting crystal growth phenomena has been suggested before [15]. Once small crystals placed near the three-phase contact line and gathered, there were gaps in the aggregate of the small crystals. Therefore, the NaCl solution was lifted up due to the capillary force and the crystallization occurred at the edge of the aggregate, which was the opposite side of the solution. Therefore, the crystal growth occurred upward.

We discuss the mechanism of alternation between the wetting and drying crystals. One possibility is that it originated from the temperature dependency of the solubility. It has been reported that the oscillation of the surface temperature occurs during the evaporation of the solvent [14]. Here, a similar oscillatory behavior was reported in the case of the methanol solution of camphor [17]. In this case, the alternation between wet and dry occurs and is explained as follows: at first, the surface of the solution covered with camphor solid skin due to the evaporation of methanol, but there is a solution below the solid skin. This solid prevents the evaporation of methanol and, thus, the surface temperature increases, resulting in the solubility of the camphor to increase. Once the temperature overcomes the threshold value, the wetting process rapidly occurs and the evaporation of methanol starts again. After that, the surface temperature decreases due to the heat of vaporization and the camphor solid skin appears again. This oscillatory behavior is similar to our observation in the NaCl system. However, the solubility of NaCl was almost independent of temperature. Therefore, to verify the above mechanism for our NaCl crystal formation system, further careful experiments are required.

Another possibility is caused by the surface tension depending on the concentration. The surface tension increases with NaCl concentration [18]. Thus, concurrent with the evaporation of the solvent on a solid NaCl, the surface tension gradient was generated and the Marangoni flow enhanced the wetting process of the aggregate of NaCl. This time, the solution around the NaCl aggregate was supersaturated (wetting state). Thus, once the crystallization started, it finished in a short time (drying state). After that, the evaporation made the concentration gradient again and repeated the wetting and drying. The oscillation mechanism is still under investigation. To clarify the mechanism, it needs further experiments.

The dendrite pattern tends to be formed by a higher concentration of source solution. This result indicates that the growth speed at a higher curvature region increases with NaCl concentration. In the case of a high concentration source, crystallization rapidly occurred soon after the wetting of the solid. In particular, the evaporation at the convex region was faster than the concaved region. Thus, the growth speed at the high curvature region became fast. However, if the concentration of the source solution was low, it needed a longer time to reach the saturation concentration and to start crystallization. Thus, the solution covering the solids became flat due to the surface tension. It prevented a faster growth at the convex region. Therefore, the low concentration source produced a low fractal dimension structure.

## 5. Conclusions

We experimentally investigated the dendrite structure of NaCl produced by a simple drying of the aqueous solution. With the evaporation of water, the solid of NaCl was produced on the wall of the container and it grew upward, which was opposite direction of the NaCl solution. Our experimental results indicated that the fractal dimension of the solid NaCl structure increased with the concentration of the source solution. In addition, during the growth process, the alternation between wetting and drying was observed. Based on our observation, we suggested some possible mechanism in this paper. However, the detailed mechanism of the producing dendrite structure and alternation between the wetting and drying are still under investigation. Although it was a quite simple experiment of just drying the NaCl solution, it included complex nonlinear phenomena and realized the oscillation and dendrite pattern formation.

## Data Availability

Not applicable.

## Supplementary Materials

The following are available online at https://www.mdpi.com/article/10.3390/ma14164434/s1, Video S1: Growth process of NaCl dendrite.
